#  Agreement to build better scientific journals in Colombia: The San Francisco Declaration on Research Assessment

**Published:** 2014-12-30

**Authors:** Mauricio Palacios Gómez, Angela Maria Franco

**Affiliations:** 1 Editor Asociado, Revista Colombia Médica. Universidad del Valle, Cali, Colombia.; 2 Vicerrectora, Vicerrectoria de Investigaciones, Universidad del Valle, Cali, Colombia.

Colombian journals need to be characterized as having an editorial guideline oriented towards achieving international standards and have the best conditions to be accepted in databases, repositories, and bibliometric indices. Deployment of proposals should encompass aspects of policy research, training and technology necessary to induce a significant change in the diffusion and dissemination of knowledge. Open access principles, the use of open source, protection of intellectual property and recognition of the formation of the editor must be the basis of a policy of national publications.

The journals need to implement a medium-term strategy to improve the diffusion of national scientific production. These changes should aim to implement high-quality editorial processes, and article level metrics that measure the usage and impact of individual scholarly activities published in the country. The national bibliographic database, Publindex should encourage the use of Open Source platform for publishing Open Access Research Articles. Peer-reviewed academic journals will strengthen because these web tools will allow optimization of the editorial workflow from the receipt of the manuscript to its publication. The complexity of the editorial processes along with the demand of editing standards may encourage the use of the open-source platform (such as Open Journal System or Ambra) 1. Delay of the country to implement programs including content management mandatorily involves a loss of competitiveness in the dissemination of research. Obligation and resources must coexist to make significant progress and to achieve the required standards. 

 The use of Digital Object Identifiers, (DOI) 2 must be mandatory for the publication of articles in electronic journals. This system is inexpensive, protects and promotes intellectual property and increases the visibility of scientific production. But again, it requires a platform editorial content manager for the results of this implementation so that they are efficient and quickly transferred to any of the available three metric systems 3. These two technology elements guarantees a development strategy to journals by national agencies (e.g., Colciencias in Colombia), to makers, guide, direct, coordinate, execute and implement the state policy in the fields of science, technology, and innovation research innovation. But, these resources are worthless without a publication ethics for editors and publishers.

 The adhesion and planned implementation, with clear goals of *Code of Conduct and Best Practice Guidelines for Journal Editors and Publishers of Ethics Committee Publications*, COPE 4 is a challenge that requires specialized training for journal editors, according with the increasingly complex demands of this cargo 5. In the midterm, it is expected that this training will result in better editorial quality and scientific manuscripts.

 Finally, reactions, protests, fear and criticism in anticipation of more competitive models can affect scientific journals, institutions and researchers when changes are made to appear. A proposal to define a policy that is consistent with the needs of everyone involved should adhere to the San Francisco Declaration on Research Assessment,DORA 6; an agreement between researchers, scientific journals and scientific organizations proposed by American Society of Cell Biology, which so far has been endorsed by 547 organizations and 12,055 scientific researchers around the world. A total of 18 declaration points that engage funders, institutions, journals and researchers were defined. 

It is necessary that Colciencias and Colombian universities adhere to this initiative since it will be the roadmap for the actions required to improving the quality of national publications. The commitment of our researchers and journals will define the harmony for the modernization of the editorial process and, above all, aspire to become a model for other institutions and researchers. These actions would fulfill the mission of the University, which is transforming society.
